# Editorial: Glial cells in health and disease: impacts on neural circuits and plasticity

**DOI:** 10.3389/fncel.2025.1569725

**Published:** 2025-02-20

**Authors:** Shirin Hosseini, Poonam Thakur, David L. Cedeno, Masoud Fereidoni, Mahmoud Elahdadi Salmani

**Affiliations:** ^1^Department of Cellular Neurobiology, Zoological Institute, TU Braunschweig, Braunschweig, Germany; ^2^School of Biology, IISER Thiruvananthapuram, Thiruvananthapuram, Kerala, India; ^3^Department of Psychology, Illinois Wesleyan University, Bloomington, IL, United States; ^4^Department of Biology, Faculty of Science, Ferdowsi University of Mashhad, Mashhad, Iran; ^5^School of Biology, Damghan University, Damghan, Iran

**Keywords:** microglia, astrocytes, oligodendrocytes (OLs), Innate Lymphocyte Cells (ILCs), neurons

This Research Topic has markedly advanced the current understanding of the role of glial cells in pathological conditions of both the central nervous system (CNS) and peripheral nervous system (PNS) (Verkhratsky et al., 2014; Tan et al., 2021). The diversity of these conditions is largely dictated by the glial cells in the specific regions of the affected neural tissue and the distinct interactions between glial cells and other cells. Investigating the common molecular mechanisms underlying these conditions can illuminate pathways for the development of innovative therapeutic strategies.

This comprehensive analysis synthesizes findings from the key studies in this research area, highlighting critical intersections between neuroimmunology and cellular neurophysiology.

The study by Kleidonas et al. delineated the acute effects of elevated ammonia levels, relevant to liver dysfunction, on the synaptic transmission of CA1 neurons in mouse entorhino-hippocampal tissue. Elevated ammonia suppresses neuronal activity due to its impact on astrocyte functionality, highlighting the key regulatory role that astrocytes play in maintaining synaptic homeostasis by modulating excitatory synapses. Importantly, it was observed that the inhibition of glutamine synthetase, an astrocytic enzyme, mitigates the downregulation of excitatory transmissions, reinforcing the pivotal role of astrocytes in neuronal function.

Concurrently, the study by Gabele et al. delved into the sex-specific neurotropic effects of the influenza A virus (H7N7 strain) on mouse hippocampal cells. The authors revealed that female-derived cells exhibit a more pronounced innate immune response, marked by elevated interferon-β and chemokine secretion, leading to differential microglial activation and morphological neuronal changes compared to male-derived cells. This sex-specific variation in immune response highlights the intricate dynamics of pathogen or stressor-host interactions within the CNS, which warrants careful consideration of sex-specific effects in future research endeavors (Chen et al., 2018).

The review study by Kveštak et al. extended the discussion to Innate Lymphoid Cells (ILCs), which, despite being a minor component of the CNS under physiological conditions, infiltrate the CNS parenchyma during neuroinflammatory and infectious scenarios. ILCs, through interactions with other immune cells, such as astrocytes and microglia, modulate CNS tissue responses, thereby influencing the outcome of autoimmune diseases and viral infections. This understanding of ILC function aligns with the findings of the previous two studies suggesting potential synergistic effects between cellular stress responses of the peripheral immune system and immune modulation in the CNS.

The interactions between glial cells and neurons in the PNS play a critical role in addition to those in the CNS (Müller et al., 2025). The study by Mohamed et al. added to our understanding of the multifaceted roles of protein kinase C epsilon type (PKCε) within the PNS. While PKCε is already recognized for its involvement in cancer progression, this study uncovered a novel function in Schwann cells (SCs), the glial cells of the PNS that are crucial for peripheral nerve regeneration and functionality. This work elucidated the ability of PKCε to modulate SC proliferation, migration, and differentiation, highlighting its role in facilitating the transition between proliferative and differentiated states, along with an epithelial-mesenchymal transition-like process. The identification of a BDNF-TrkB-PKCε autocrine signaling axis provided valuable insights into the molecular mechanisms regulating SC plasticity. This groundbreaking work suggests that targeting this pathway may open new therapeutic avenues for addressing peripheral nerve injury, neuropathic pain, peripheral nerve tumors, and related neuropathies, thereby broadening the scope of intervention for PNS-related diseases.

In the work conducted by Schröder et al., a rational drug design approach was employed to address Multiple Sclerosis (MS), a chronic, autoimmune, demyelinating disease of the CNS; although clinical studies have demonstrated PNS involvement in a subgroup of patients (Adamec et al., 2021). The study focused on polysialic acid (polySia), a glycan recognized for its immunomodulatory properties, demonstrating its potential benefits in the upregulation of myelin. Notably, the application of polySia with a chain length of DP24–30 in organotypic murine brain slices was found to significantly enhance remyelination. This effect is mediated by modulation of microglial activation via the inhibitory immune receptor Siglec-E. Furthermore, the findings indicate that polySia effectively reduces proinflammatory responses in microglia, fostering an environment that supports myelin regeneration. By elucidating the immunomodulatory and pro-regenerative capabilities of polySia, this study contributes to a deeper understanding of the mechanisms underlying myelin repair, establishing polySia as a promising therapeutic target for treating demyelinating diseases such as MS.

Remarkably, the study of polySia on SCs is particularly intriguing, as polySia has been shown to enhance SC proliferation and migration, thereby promoting peripheral nerve regeneration and myelination, which may offer novel therapeutic insights for neuropathic conditions and nerve injury.

In the mini-review by Gjervan et al., an alternative strategy for myelin repair was presented that focused on claudin-11, a critical protein of tight junctions within the myelin sheath. Recent studies have elucidated that heterozygous stop-loss mutations in the *CLDN11* gene are implicated in the etiology of hypomyelinating leukodystrophy 22 (HLD22), a genetic disorder characterized by impaired myelination. Investigations conducted in claudin-11-deficient mouse models have demonstrated significant physiological impairments, such as delayed nerve transmission, auditory deficits, and male sterility, thereby revealing the multifaceted roles of this protein. These findings underscore the urgency of further research aimed at elucidating the full spectrum of claudin-11′s functions and advancing the development of effective therapeutic interventions, not only for HLD22 and related conditions but also for other demyelinating diseases.

**Figure 1:**
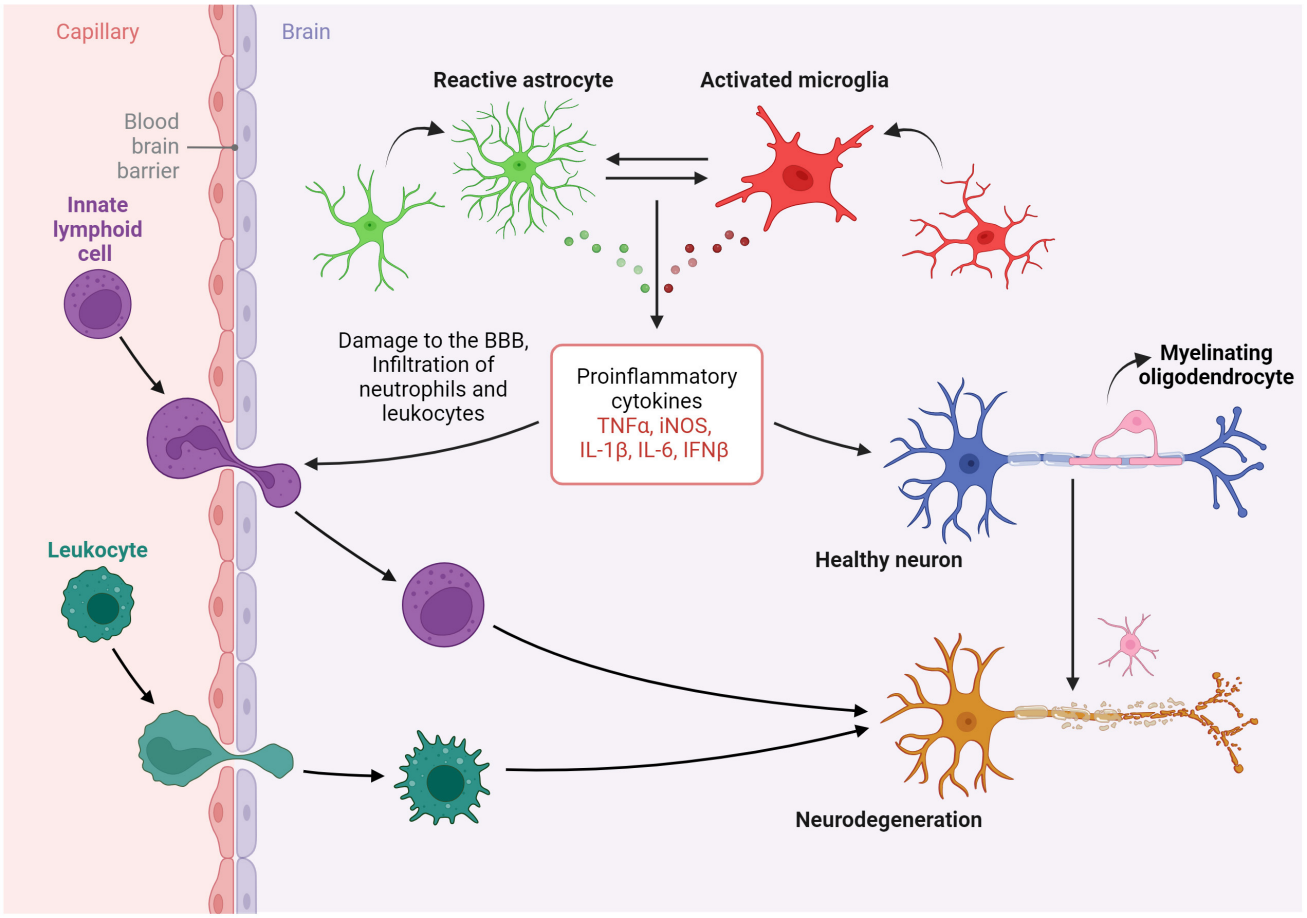
The evidence elucidates the distinctive interactions among glial cells, peripheral immune cells, and neurons in response to various stressors that are shaped by inherent sex-based differences. This integrative perspective significantly advances our understanding of pathophysiology in both the peripheral and central nervous systems, particularly with regard to synaptic regulation, immune cell interactions, and the development of potential therapies for conditions of the nervous system in the context of neuroinflammatory conditions. Created in BioRender (https://BioRender.com/h64r139).

Taken together, the articles in this Research Topic underscore a nuanced paradigm in which glial cells, peripheral immune cells, and neurons interact differentially under metabolic, pathologic and infectious stressors, influenced by inherent sex-based immunological differences (Figure 1). This integrative perspective advances our understanding of PNS and CNS pathophysiology, particularly in the elucidation of mechanisms underlying synaptic regulation, immune cell interactions, and the development of potential therapeutic interventions targeting nervous system disorders in the context of acute and chronic neuroinflammatory conditions.
